# Acculturation and 4-year caries increment among children of foreign-born mothers in Sweden: a register-based cohort study

**DOI:** 10.1186/s12903-022-02130-4

**Published:** 2022-04-07

**Authors:** Anna Granlund, Fernanda Cunha Soares, Anders Hjern, Göran Dahllöf, Annika Julihn

**Affiliations:** 1 Public Dental Service, Folktandvården Stockholms Län AB, Stockholm, Sweden; 2 Center for Pediatric Oral Health Research, Stockholm, Sweden; 3 Division of Orthodontics and Pediatric Dentistry, Department of Dental Medicine, Karolinska Institutet, Stockholm, Sweden; 4 Clinical Epidemiology, Department of Medicine, Karolinska Institutet, Stockholm, Sweden; 5 Centre for Health Equity Studies (CHESS), Stockholm University, Stockholm, Sweden; 6 Center for Oral Health Services and Research, Mid-Norway, Trondheim, Norway; 7 Department of Pediatric Dentistry, Eastmaninstitutet, Folktandvården Stockholms Län AB, Stockholm, Sweden

**Keywords:** Acculturation, Child, Dental caries, Ethnicity, Human development index, Length of stay, Migration, Socioeconomic status

## Abstract

**Background:**

To study the association of maternal age upon arrival and length of residence in Sweden with the 4-year caries increment in their children between ages 3 and 7 years in relation to the human development index (HDI) of the maternal country of origin.

**Method:**

This registry-based cohort study included all children born in 2000–2003 who resided in Stockholm County, Sweden, at age 3 years and who were followed up at age 7 (*n* = 63,931). Negative binomial regressions were used to analyze different models adjusted for sociodemographic factors.

**Results:**

Children of foreign-born mothers, regardless of the HDI of the maternal country of origin, had a higher risk of caries increment between ages 3 and 7 years than children of Swedish-born mothers. Furthermore, children of mothers who had arrived from a low or medium HDI country had a lower caries increment if their mothers arrived before age 7 compared with after age 7. Nearly half (44%) of the children whose mothers arrived in Sweden at age ≥ 20 years from a low HDI country had a caries increment compared to 22% of the children whose mothers had arrived in Sweden before 7 years of age. Furthermore, children whose mothers were born in a low HDI country and had resided in Sweden ≤ 19 years had approximately 1.5 times higher risk of caries increment compared to children of mothers who had resided in Sweden for more than 20 years.

**Conclusions:**

Caries increment in the children of foreign-born mothers was associated with the age of their mother when she arrived in Sweden and was lower when the mother had arrived before age 7 years. This indicates an intergenerational effect that carries over to the children and is greater the longer the mother has participated in Swedish dental healthcare.

## Background

Global migration has increased dramatically. In 2019, the number of migrants reached an estimated 272 million globally, 51 million more than in 2010; international migrants comprised 3.5% of the global population [1]. In Sweden, approximately 20% of the population have a foreign background [2]. Of these, over 800,000 are children and adolescents who were born in another country or were born in Sweden to one or two foreign-born parents [3].

Sweden, like most developed countries, has seen a decrease in dental caries among children and adolescents in recent decades, but there are still groups of children with high caries experience [4]. A study in the north of Sweden reported that 58% of 4-year-olds with an immigrant background had dental caries compared to 15% of Swedish children [5]. Naturally, several socioeconomic factors may explain this difference. Low family income and maternal education, age at childbirth, single parenthood, and maternal health behaviors such as smoking and obesity during pregnancy have all been significantly linked to caries experience at 3 and 7 years of age [6, 7]. A recent study reported that, compared with children born to two Swedish parents, children in Sweden born to foreign-born parents from low and medium human development index [8] countries had the highest caries experience at 3 and 7 years of age, and from high HDI countries, a significantly higher caries experience. The study also found that children in families with a mixed background had a significantly higher risk of caries experience compared to children with two native Swedish parents [9]. Immigrants in Sweden with a non-European background report that they suffer from poor or very poor general health three to four times more often than the native Swedish population [10].

The migration process involves changes that will affect the lifestyle and health behaviors of those making their home in the new country. Children with foreign-born background, have been reported to have a higher intake of ice cream, sweets and chocolate drinks and a lower frequency of tooth-brushing compared with children without foreign-born background [5]. In addition, a previous study showed that adults in minority populations in Sweden use less dental care less frequently even though having a greater need of dental treatment [11].

The process of adopting values and behaviors from another culture, and perhaps in time, also changing their beliefs, is referred to as acculturation [12]. A review of the oral health impacts of acculturation [13] concluded that information is limited and fragmented. Several studies have shown positive associations between acculturation indicators and use of dental services and better oral health among acculturated individuals [14]. On the other hand, acculturation may also promote behaviors that negatively affect oral health, such as the introduction of a more cariogenic diet [15].

Previous studies on acculturation in relation to dental caries in children of foreign-born parents have a cross-sectional design, they have combined together immigrants from different parts of the world with different experiences and often lack information on the current socioeconomic situation [16]. Thus, the aim of this study was to investigate the association between maternal acculturation and caries increment between 3 and 7 years of age in children of foreign-born women in Sweden.

## Methods

### Study design

The present study is a longitudinal study and part of a register-based cohort study on development of dental caries in children between 3 and 7 years of age. Data sources were based on Swedish national registry data held by the Swedish National Board of Health and Welfare and by Statistics Sweden (SCB). Information on dental caries was collected from data sources at the Public Health Care Administration in Stockholm.

### Study population

The study population was created from the population of child residents in Stockholm County, born in 2000–2003 (n = 83,147). The present study evaluated children who had been examined at 3 and 7 years of age in the Public Dental Service, by private practitioners, or at the Department of Dental Medicine, Karolinska Institutet. When the mother was foreign-born, participants were excluded from the study if there was no information on the maternal country of origin, age upon arrival in Sweden, or length of residence in Sweden. A total of 83,147 children aged 3 years were evaluated. At 7 years of age, the follow-up rate was 77% (n = 63,931). The children at follow-up showed similar characteristics as the children at baseline (P < 0.05); therefore, study dropout did not affect the descriptive characteristics of the sample.

### Independent variables

Information on age, year of arrival, and maternal country of origin was obtained from the Swedish Registry of Total Population at SCB. We classified countries into four categories for maternal nationality; the first category was Swedish-born mothers. For the other categories we used the HDI developed by the United Nations Development Program. It is a composite index of life expectancy, education level, and per capita income indicators. We used the HDI from 1999, the year before the children in this study were born (HDI 1999). The HDI cut-off values were as follows: low = 0.350–0.449, medium = 0.450−0.699, and high ≥ 0.700 [8], (Table 1).

**Table 1:** Countries of origin of the mothers of 63,931 children who participated in the study

Maternal country of origin		
Category	n	Country (% in category)
*Non-immigrant*	49,503	Sweden (100%)
*Immigrant*	14,428	
High HDI	2938	Western Europe (18.1%), Nordic countries (53.4%), Southern Europe (6.9%), North America (12.0%), Korea (9.6%)
Medium HDI	8974	Eastern Europe (32.5%), South America (12.5%), China (2.2%), Other Asia (51.9%), Vietnam (0.5%), Oceania (0.4%)
Low HDI	2516	Africa (92.9%), India (7.1%)

*HDI* human development index; cut-offs: low = 0.350–0.449, medium = 0.450−0.699, high ≥ 0.700

From the database of The Swedish National Board of Health and Welfare, which maintains the Swedish Medical Birth Registry (MBR), we collected maternal age at childbirth (14–20 years; 21–25 years; 26–30 years; 31–35 years; > 35 years) and family situation (single mothers, cohabitants). The SCB determines disposable household income using an algorithm that considers all household incomes reported to the Swedish Tax Agency, reduced by all taxes, and then divided by consumer units. The range of disposable household income in the study was used by first constructing quintiles and thereafter categorizing them as low (1st and 2nd quintiles (≤ 1162 SEK)), medium (3rd quintile (> 1162 and ≤ 1493 SEK)), and high (4th and 5th quintiles (> 1493 SEK)). Maternal educational level was classified as ≤ 12 years and > 12 years of education.

Acculturation proxy measures were evaluated by “maternal age upon arrival in Sweden”, classified as < 6, 7 − 12, 13 − 19, and ≥ 20 years of age, and “length of maternal residence in Sweden at childbirth” was calculated as the difference between the child’s birth year and the year that the mother had arrived in Sweden.

### Outcome variable

In Stockholm county, children usually get an oral examination every 2 years, i.e. at 3, 5 and 7 years of age for young children. However, since data on dental caries, according to the deft indices in young children, only are sent at 3 and 7 years from Public Dental Service, by private practitioners, or at the Department of Dental Medicine, Karolinska Institutet to the Public Health Care Administration in Stockholm, we only have caries data at these ages.

Data on manifest caries lesions were collected from clinical and radiographic examinations. The decayed, extracted, and filled primary teeth (deft) index measured the severity of caries experience in children at 3 and 7 years of age. The definition of “manifest caries” as a lesion that clearly extends into the dentin was used in this study [17]. Radiographic examinations in the form of bitewings were only done given indications, to provide more extensive data on dentin caries lesions. The variable caries increment (Δ deft) was defined as the difference between deft at age 3 and deft at age 7 years; the result was then dichotomized as “no caries increment" (Δ deft = 0) or “caries increment” (Δ deft ≥ 1). No permanent teeth were included in the outcomes at 7 years of age.

### Statistical analyses

STATA 14 for Windows (Stata Statistical Software; StataCorp LP; College Station, TX, USA) was used for data analysis. Descriptive analyses included relative and absolute frequencies. Differences between categorical variables were assessed using the chi-square test. We used negative binomial regressions to analyze 10 models, estimating incidence rate ratios (IRR) with 95% confidence intervals to assess the association between caries increment in children between 3 and 7 years of age (0: no risk group; 1: risk group). We modeled X by Y, being Y caries increment and X independent variables that varied according to the models below (Table 2):

**Table 2:** Path of negative binomial analysis to assess associations between caries increment in children (3–7 years)

Model	Sample	Y	X	Reference group	Adjustment
I	All children	Caries increment in children between 3 and 7 years of age (0: no risk group; 1: risk group)	HDI of the maternal country of origin concurrently with maternal age upon arrival in Sweden	Swedish-born population	Sex, maternal age, family situation, income
II	Children whose mothers born in low HDI countries		Maternal age upon arrival in Sweden	Mothers who arrived in Sweden before age 7 years	Sex, maternal age, family situation, income
III			Length of residence in Sweden of mothers	Mothers who had lived in Sweden for 20 years or more	
IV			Maternal age upon arrival in Sweden	Mothers who arrived in Sweden before age 7 years	Sex, maternal age, family situation, income, length of residence in Sweden
V	Children whose mothers born in medium HDI countries		Maternal age upon arrival in Sweden	Mothers who arrived in Sweden before age 7 years	Sex, maternal age, family situation, income
VI			Length of residence in Sweden of mothers	mothers who had lived in Sweden for 20 years or more	
VII			Maternal age upon arrival in Sweden	Mothers who arrived in Sweden before age 7 years	Sex, maternal age, family situation, income, length of residence in Sweden
VIII	Children whose mothers born in high HDI countries		Maternal age upon arrival in Sweden	Mothers who arrived in Sweden before age 7 years	Sex, maternal age, family situation, income
IX			Length of residence in Sweden of mothers	Mothers who had lived in Sweden for 20 years or more	
X			Maternal age upon arrival in Sweden	Mothers who arrived in Sweden before age 7 years	Sex, maternal age, family situation, income, length of residence in Sweden

We used the Bayesian Information Criterion (BIC) to assess the overall fit of a model and compare various models.

## Results

Of the 63,931 eligible children who were evaluated, 23% had a foreign-born mother. Table 3 presents the categories of maternal country of origin. At childbirth, 36% of the foreign-born mothers had resided in Sweden for 5 years or less while 25% had resided in Sweden for more than 15 years. Twenty percent of the foreign-born mothers came from low HDI countries and 62% from medium HDI countries. Compared with Swedish mothers, foreign-born mothers were more likely to have a lower income (62% vs 31%) and a lower educational level. They were also more likely to have given birth before they had reached 20 years of age (Table 3).

**Table 3:** Sociodemographic characteristics of mothers who resided in Stockholm County and gave birth in 2000–2003

Variables	Category	Swedish-born (n = 49, 503)		Foreign-born (n = 14, 428)	
		n	%	n	%
Income level^*^	Low	15,286	31	8907	62
	Medium	10,725	22	2276	16
	High	23,271	47	3125	22
Education level^*^ (years)	Low (≤ 12)	27,686	56	5912	41
	High (> 12)	21 817	44	8516	59
Family situation^*^	Single	8657	18	3336	23
	Cohabitant	40,846	82	11,092	77
Maternal age at childbirth^*^ (years)	14–20	700	1	435	3
	21–25	4787	10	2645	18
	26–30	16,046	32	4474	31
	31–35	19,128	39	4412	31
	> 35	8842	18	2462	17
HDI category of maternal country of origin	Low	–	–	2938	20
	Medium	–	–	8974	62
	High	–	–	2516	18
Mother’s length of residence in Sweden at childbirth (years)	≤ 5	–	–	5605	36
	6–10	–	–	3691	24
	11–15	–	–	2329	15
	> 15	–	–	3922	25

*HDI* human development index; cut-offs: low = 0.350–0.449, medium = 0.450−0.699, high ≥ 0.700. ^*^p < 0.001

Table 4 presents the proportion of children who developed dental caries between 3 and 7 years of age in relation to maternal age upon arrival in Sweden and HDI of the maternal country of origin. Among mothers from a low or medium HDI country of origin, the proportion of 7-year-olds with a 4-year caries increment was lower when the mother had arrived before 7 years of age compared with arriving at an older age. Among mothers from a low HDI country of origin, the caries increment of children of mothers who had arrived in Sweden at age 20 years or older was 44% compared to 22% caries increment of children whose mothers had arrived before age 7. In contrast, this gradient did not occur in children of mothers from high HDI countries; that is, maternal age upon arrival in Sweden had non-significant bearing on the development of a caries increment in their children.

**Table 4:** Caries increment between 3 and 7 years of age in relation to maternal background

Maternal background	Caries increment^a^				Total	
	Δ deft = 0		Δ deft ≥ 1			
	n	%	n	%	n	%
*Non-immigrant*						
Swedish-born	40,792	82.4	8711	17.6	49,503	100
*Immigrant (HDI of country of origin and age upon arrival in Sweden [years])*						
Low HDI						
0–6	90	78.3	25	21.7	115	100
7–12	61	64.2	34	35.8	744	100
13–19	241	59.5	164	40.5	405	100
≥ 20	1042	56.0	820	44.0	1862	100
Total	1434	57.9	1043	42.1	2477	100
Medium HDI						
0–6	509	66.2	260	33.8	769	100
7–12	442	58.1	319	41.9	761	100
13–19	738	47.7	809	52.3	1547	100
≥ 20	2948	51.0	2832	49.0	5780	100
Total	4637	52.4	4220	47.6	8857	100
High HDI						
0–6	691	75.9	219	25.1	910	100
7–12	168	81.2	39	18.8	207	100
13–19	167	70.2	71	29.8	238	100
≥ 20	1204	78.9	322	21.1	1526	100
Total	2230	77.4	651	22.6	2881	100

^a^Caries increment: The difference between deft at age 3 and deft at age 7 (∆ deft) was dichotomized as “no caries increment" (∆ deft = 0) or “caries increment” (∆ deft ≥ 1). No permanent teeth were included in the outcomes at age 7. *HDI* human development index; cut-offs: low = 0.350–0.449, medium = 0.450−0.699, high ≥ 0.700

**Fig. 1:**
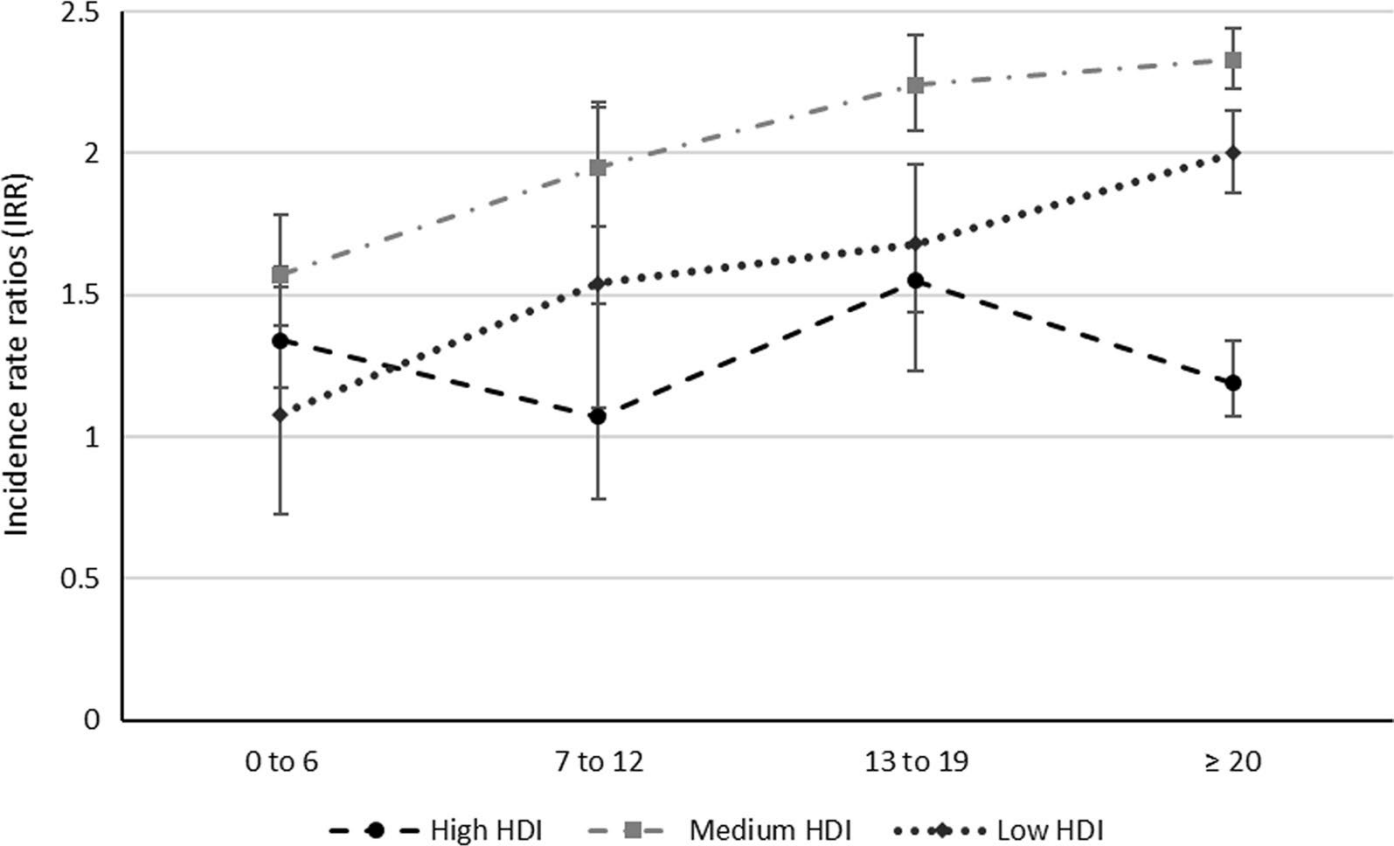
Association (incidence rate ratio) of age of foreign-born mothers upon arrival in Sweden and their children’s risk of developing a caries increment between ages 3 and 7 years with the HDI of the maternal country of origin using Swedish-born mothers as a reference. Logistic regression analyses were adjusted for length of maternal residence in Sweden, sex of child, maternal age at childbirth, family situation, and income (HDI, human development index; cut-offs: low = 0.350–0.449, medium = 0.450−0.699, high ≥ 0.700

Figure 1 shows that, independent of the HDI of the maternal country of origin, children of foreign-born mothers had a significantly higher risk of caries increment between ages 3 and 7 compared to children of Swedish-born mothers. More specifically, when compared to children of Swedish mothers, children whose mothers were born in low (IRR: 2.00; 95% CI: 1.86–2.15) or medium (IRR: 2.33; 95% CI: 2.23–2.44) HDI countries and had arrived in Sweden at age 20 years or older had the highest risk of caries increment. The children of mothers from low and medium HDI countries exhibited a marked decrease in caries risk if the mothers had arrived before age 7. The children of mothers born in medium HDI countries had an IRR of 1.57 for caries increment (95% CI:1.39–1.78) while the IRRs of children of Swedish-born mothers and of children of mothers born in low HDI countries differed non-significantly.

Table 5 shows the IRRs for the 4-year caries increment (Δ deft ≥ 1 between ages 3 and 7 years) in children of foreign-born mothers in relation to maternal age upon arrival and length of stay in Sweden. We used three models. Model I assessed the risk of caries increment, using as a reference children of mothers who had arrived in Sweden between 0 and 6 years of age, and adjusted for sex of the child, maternal age, family situation, and income. Children of mothers from both low and medium HDI countries who had arrived in Sweden after 13 years of age had around a 1.5 times higher risk of caries increment compared to children of mothers who had arrived before 7 years of age. Maternal age upon arrival in Sweden was unrelated to caries increment in children of mothers from high HDI countries, having no effect on caries risk.

**Table 5:** Caries increment according to maternal age upon arrival and length of stay in Sweden

HDI of maternal country of origin and age upon arrival in Sweden (years)	Reference								
	Maternal age upon arrival in Sweden (0–6 years)			Maternal length of stay in Sweden (≥ 20 years)			Maternal age upon arrival in Sweden (0–6 years)		
	n	IRR (95% CI)	*p*	n	IRR (95% CI)	*p*	n	IRR (95% CI)	*p*
*Low HDI*	*Model I(a)* *BIC’* = *68.65*			*Model II(a)* *BIC’* = *71.62*			*Model III(a)* *BIC’* = *75.86*		
0–6	115	1	–	1 199	1.52 (1.10:2.10)	0.011	115	1	–
7–12	95	1.51 (0.90:2.54)	0.120	844	1.44 (1.04:1.99)	0.030	95	1.55 (0.90:2.66)	0.113
13–19	405	1.65 (1.08:2.53)	0.021	241	1.56 (1.09:2.24)	0.016	405	1.73 (1.04:2.88)	0.036
≥ 20	1862	1.83 (1.22:2.74)	0.003	163	1	–	1832	1.95 (1.10:3.45)	0.022
*Medium HDI*	*Model I(b)* *BIC’* = *− 82.60*			*Model II(b)* *BIC’* = *− 72.97*			*Model III(b)* *BIC’* = *− 75.69*		
0–6	769	1	–	4311	1.30 (1.17:1.45)	< 0.001	769	1	–
7–12	761	1.25 (1.06:1.47)	0.008	2135	1.29 (1.15:1.45)	< 0.001	761	1.22 (1.02:1.45)	0.027
13–19	1547	1.44 (1.25:1.65)	< 0.001	1239	1.21 (1.06:1.37)	0.005	1547	1.36 (1.13:1.64)	0.001
≥ 20	5780	1.43 (1.26:1.63)	0.002	1117	1	–	5725	1.31 (1.05:1.65)	0.018
*High HDI*	*Model I(c)* *BIC’* = *50.41*			*Model II(c)* *BIC’* = *51.93*			*Model III(c)* *BIC’* = *57.76*		
0–6	910	1	–	863	0.87 (0.72:1.05)	0.147	910	1	–
7–12	207	0.84 (0.58:1.21)	0.345	513	0.88 (0.70:1.10)	0.268	207	0.84 (0.58:1.21)	0.345
13–19	238	1.25 (0.83:1.87)	0.286	321	1.03 (0.81:1.32)	0.810	238	1.25 (0.83:1.87)	0.286
≥ 20	1526	1.05 (0.62:1.77)	0.867	1158	1	–	1500	1.05 (0.62:1.77)	0.867

*HDI* human development index; cut-offs: low = 0.350–0.449, medium = 0.450−0.699, high ≥ 0.700; BIC’, Bayesian Information Criterion; In bold: the best model (smallest BIC’); Model I—adjusted: sex, maternal age at childbirth, family situation, income; Model II—adjusted: sex, maternal age at childbirth, family situation, income; Model III—adjusted: mother’s length of stay in Sweden at childbirth, sex, maternal age at childbirth, family situation, income

Model II assessed the risk of caries increment in children in relation to maternal length of residence in Sweden with 20 years or more as a reference and adjusted for sex of the child, maternal age, family situation, and income. Children of mothers born in low HDI countries who had resided in Sweden for 19 years or less had around a 1.5 times higher risk of caries increment compared to children of mothers who had resided in Sweden for more than 20 years. Risk decreased to about 1.3 in children of mothers born in medium HDI countries. Children of mothers from high HDI countries had no increase in caries risk.

Model III presents the risk of caries increment in children of mothers who had arrived in Sweden between 0 and 6 years of age as reference, adjusted by maternal length of residence in Sweden, in addition to the other adjustments noted above. The risk of caries increment was significantly higher in children of mothers for medium HDI countries older than 7 years upon arrival in Sweden, whereas the risk for caries increment was highest in children of mothers from low HDI countries who had arrived in Sweden after 12 years of age.

Regardless of the HDI of the country of origin, the models with the best fit were the ones that took into account maternal age upon arrival in Sweden and were adjusted by sex of the child, maternal age, family situation, income.

## Discussion

The results of the present study show that children of foreign-born mothers in Sweden from the various HDI countries had a higher risk of caries increment between 3 and 7 years of age than same-aged children of Swedish-born mothers. Further, the younger the mothers were upon arrival in Sweden, the lower the risk of caries increment in their children was when maternal country of origin had a low or medium HDI.

The main finding of the present study is that the age of the mothers upon arrival in Sweden is a main determinant of caries risk in their offspring, independent of the time they had lived in Sweden before childbirth. This suggests that maternal participation in the Swedish preventive dental health program has intergenerational effects that carry over to their offspring, with a greater effect the longer the mother has participated in this program. At the time when the parents of the children in this study were children themselves, they participated in a dental health program that highly valued pediatric oral health through a focus on prevention, bi-annual dental examinations, and treatment free of charge.

About 18% of children born in families with two Swedish parents developed dental caries between 3 and 7 years of age; all immigrant groups had a significantly higher caries increment. Immigrants in Sweden, and their children, have also been found to have higher risks of general health problems, including psychiatric morbidity [18, 19] and poor subjective health [10]. Several studies have reported higher caries experience in preschool children with immigrant background [5, 11, 20]. A cross-sectional study of caries experience in Denmark found that the mean caries experience was three to four times higher among 5- and 7-year-old children with non-Danish mothers than among children of Danish-born mothers [6]. In addition to socioeconomic factors, significantly fewer foreign-born parents brush their children’s teeth twice daily with fluoride toothpaste [6], and a study on dietary transition among minority populations in the Nordic countries found a higher intake of sugary products among immigrant children aged 0–7 years [21]. In the new country, immigrants tend to adopt not only positive but also negative behaviors, which may lead to a worse health outcome than in their home countries [22]. Önal [23] found that the caries prevalence of Turkish children who were living in Turkey and had a low socio-economic status was lower than in Turkish children living in Germany or in German children; the deterioration in oral health after immigration was the result of adapting to western meals and dietary habits without improving oral hygiene.

We found that a higher proportion of children whose parental country of origin was a medium HDI country had a caries increment compared to when the parental country of origin was a low HDI country. This may seem somewhat contrary to findings reporting a gradient of health outcomes in children of immigrants related to increasing HDI of the country of origin [24, 25]. The HDI is a composite index of life expectancy, gross national product, and educational level of the population [26]. In a recent study on African countries, no difference in early childhood caries in 3–5-year-old children was found between low- and medium-income countries. It was concluded that child health and child oral health is more dependent on how society organizes itself and how it uses the resources rather than on wealth per se [27]. Most of the mothers in the medium HDI group came from eastern and central Europe where prevalence of early childhood caries is high and oral health priorities in national preventive policies are low [28].

Proxy measures of acculturation include ethnic identification, language skills, age at immigration, length of residence in the host country, and social affiliation [13, 14]. The present study used age upon arrival in Sweden as a proxy measure of acculturation, but also adjusted for length of stay in Sweden in the analyses. Adolescents who were second generation immigrants or who had arrived before 1 year of age had a caries prevalence similar with those of native Swedish adolescents, whereas those who arrived after 7 years of age had a caries prevalence that was 2–3 times higher [29]. Werneck et al. [30] also demonstrated that arrival in the new country at an early age is beneficial for the oral health of the offspring; their study among Portuguese-speaking immigrants in Canada found that children of mothers who were older than 22 when they arrived in Canada had a 2.36 times higher odds of early childhood caries compared to those whose mothers had arrived at a younger age.

In mothers from high HDI countries, maternal age upon arrival in Sweden had no significant influence on the development of a caries increment in their children. These mothers came from western and southern Europe and north America where access to preventive dental care is emphasized and available.

Berry [31] introduced the concept of acculturation strategies and suggested that this process has four possible outcomes: assimilation (when individuals do not wish to maintain their cultural identity and seek daily interaction with other cultures), separation (when individuals place a value on holding on to their original culture, and at the same time wish to avoid interaction with others), integration (when there is an interest in not only maintaining one’s original culture but also interacting daily with other groups), or marginalization (when there is little possibility or interest in cultural maintenance and little interest in having relations with others). When using this approach to study oral health among Pacific immigrants to New Zealand, Schluter et al. [32] found that while Pacific children were more likely to have poorer oral health than their non-Pacific peers, differences in cultural alignment appeared to have only a relatively modest impact against the complex backdrop of individual, social, environmental, and political conditions. We previously showed that within each stratum of mothers from low and medium HDI countries, there is a gradient of an increasing proportion of children with a caries increment between 3 and 7 years of age among those of low family income [9].

One major advantage of register-based longitudinal studies is the low attrition rate. Over 4 years, only 14% of the participants were lost to follow-up, minimizing the risk of selection bias. In clinical randomized controlled trials on caries prevention, an attrition rate of 25% over 2 years is not uncommon, particularly in high-risk populations. Also, the quality of socioeconomic data is better than of self-reported data, since they are retrieved from tax returns with reports of income and national registries that record occupation, educational level, housing, and area of residence. A limitation of this study is that asylum seeking, and undocumented children were excluded since they are not included in the national registries used.

Stockholm County is characterized by a high proportion of immigrants in its population and a high degree of housing segregation where immigrants tend to cluster into certain neighborhoods. Thus, it is possible that the pace of acculturation is higher in regions where immigrants live more integrated with the majority population.

The persistence of disparities in the dental health of children of mothers arriving in Sweden at older ages suggests that universal dental health coverage may not be sufficient in this population for ensuring appropriate behaviors regarding diet and tooth brushing twice daily with fluoride toothpaste as well as utilization of primary and preventive care. This is particularly worrisome since the fluoride-based methods used in high-risk populations now seem ineffective [33, 34]. Thus, a new clinical research agenda is needed to address this issue. We need to further explore possibilities of collaboration between pediatric healthcare and child dental care and methods of long-term support for immigrant families.

## Conclusion

The present study shows that children of foreign-born mothers from countries of all HDI levels had an increased risk of caries increment between 3 and 7 years of age. The risk of caries increment was significantly lower in children whose mothers had arrived in Sweden from low and medium HDI countries before 7 years of age. This pattern was not seen in children of mothers from high HDI countries.

## Data Availability

The data that supports the findings of this study is available from the Swedish National Board and Welfare, restriction apply to the availability of these data, it was obtained under license and therefore not publicly available. Information on dental caries was collected from data sources at the Public Health Care Administration in Stockholm. Questions or requests concerning this data is directed to author, Annika Julihn.

## Abbreviations

HDI: Human Development Index
CI: Confidence interval
deft: decayed, extracted, filled, teeth
MBR: Medical Birth Registry
OR: Odds ratio
SCB: Statistics Sweden
IRR: Incidence rate ratios
BIC: Bayesian Information Criterion
